# Current methods and mechanisms for animal models of pelvic inflammatory disease: a review

**DOI:** 10.3389/fvets.2026.1843100

**Published:** 2026-05-01

**Authors:** Guanglong Wang, Suo Zhang

**Affiliations:** College of Traditional Chinese Medicine, Inner Mongolia Medical University, Hohhot, China

**Keywords:** animal models, chemical induction, pathogen induction, pelvic inflammatory disease (PID), physical induction

## Abstract

Pelvic inflammatory disease (PID) is a complex multifactorial infectious disorder of the female reproductive tract, associated with severe long-term sequelae including infertility, ectopic pregnancy, and chronic pelvic pain, as well as elevated risks of endometriosis, cardiometabolic diseases, and colorectal cancer. Owing to ethical constraints on human research, animal models have become indispensable tools for investigating PID pathogenesis, evaluating therapeutic interventions, and developing novel diagnostic strategies. This review systematically summarizes current advances in PID animal model construction, with a focus on three core induction categories: pathogen, chemical, and physical induction methods. Pathogen induction utilizes single or multiple microorganisms (including *Escherichia coli*, *Staphylococcus aureus*, *Chlamydia trachomatis*, and *Ureaplasma urealyticum*) to recapitulate the infectious etiology of clinical PID. Chemical induction employs agents such as phenol mucilage, hydrochloric acid combined with lipopolysaccharide, and exogenous estrogen to simulate inflammatory processes via direct tissue damage or immune modulation. Physical induction methods include mechanical injury to disrupt mucosal barriers and foreign body implantation to mimic intrauterine device-related chronic inflammation. We further analyze integrated induction strategies that combine multiple approaches to improve model stability and pathological fidelity, and compare the strengths, limitations, and applicable scenarios of each modeling method. Finally, we discuss current gaps in PID animal model research, including the lack of standardized protocols, insufficient characterization of chronic disease progression, and limited translational relevance to human disease, and propose priorities for future model development to support preclinical research on PID prevention and treatment.

## 1 Introduction

Pelvic inflammatory disease (PID) is a complex, multifactorial infectious disorder (1), encompassing a spectrum of upper female reproductive tract pathologies including endometritis, salpingitis, tubo-ovarian abscess, and pelvic peritonitis (2). It leads to severe reproductive sequelae such as infertility, ectopic pregnancy, and chronic pelvic pain, all of which significantly impair women’s quality of life (3), and is associated with an increased risk of endometriosis, hypertension, diabetes, and colorectal cancer (4–8). Epidemiologically, 33.6% of British women aged 35–44 have had at least one PID episode, 16.1% have a history of salpingitis, and 2 million childbearing-aged women in the United States self-reported a PID history (9). Among 4 nationally representative US datasets, 3 showed an overall decline in self-reported PID history and PID-related emergency/outpatient visits, yet nearly all sources have recorded a slight rebound since 2015 (10). Global age-standardized rates of PID and ectopic pregnancy (EP) presented a mild downward trend from 1990 to 2019, but both conditions remain major public health challenges with a strong intercorrelation (11). Despite advances in modern diagnostic and therapeutic strategies, PID remains a leading global cause of the aforementioned reproductive sequelae (1, 12). Its intricate pathogenesis involves interactions between polymicrobial infection, host immune responses and inflammatory signaling cascades, highlighting the urgent need for in-depth research into its underlying mechanisms (13, 14). However, ethical constraints on human research and the inherent complexity of PID necessitate the use of animal models to investigate its pathophysiology, evaluate potential therapeutics and develop novel diagnostic tools.

Animal models are essential in PID research, as they recapitulate human PID pathological features in a controlled setting, allowing systematic exploration of disease pathogenesis, inflammatory mediator function and pathogen-host interactions, and are widely applied to evaluate the efficacy and safety of antibiotics, anti-inflammatory drugs and other therapies to promote the bench-to-clinic translation of new treatments (15–17). Published reviews on PID animal models are scarce, mostly focusing on individual model establishment and validation. A systematic classification of their types, construction strategies, and application values is thus urgent. This review summarizes current progress, highlights key technical details (model establishment, reagent selection, experimental design), and elaborates on the pathological mechanisms of different construction methods, to provide a reference for future research.

## 2 The pathogenesis of PID

The pathogenesis of PID was first elucidated approximately 50 years ago (18). A clear understanding of PID pathogenesis is critical for the rational design of animal models. The core pathogenic mechanism of PID can be summarized as follows: mixed infections caused by exogenous sexually transmitted pathogens [primarily *Neisseria gonorrhoeae*, *Chlamydia trachomatis*, and *Mycoplasma genitalium* (19)] and endogenous commensal opportunistic pathogens in the vagina [such as aerobic and anaerobic bacteria (20, 21)] serve as the primary initiating factors; mediated by predisposing factors such as impaired cervical mucosal barrier function and reduced innate and adaptive immune defenses, the pathogens ascend along the reproductive tract mucosa, breach the cervical mechanical and immune defense barriers, and sequentially invade and colonize the endometrium, fallopian tubes, ovaries, and pelvic peritoneum, as well as other upper female reproductive tract and surrounding tissues. These predisposing factors include multiple high-risk factors for PID, including intrauterine procedures such as induced abortion and intrauterine device (IUD) insertion (22), anatomical abnormalities of the reproductive tract (23–25), spread of inflammation from adjacent organs (26), and high-risk sexual behaviors such as having multiple sexual partners and unprotected sex (27). A persistent, uncontrolled inflammatory response can lead to the destruction and sloughing of the ciliated epithelium of the fallopian tubes, mucosal necrosis, tissue edema, and abscess formation, ultimately progressing to tubal adhesions and obstruction, extensive pelvic fibrosis and adhesions, and resulting in irreversible long-term complications such as tubal infertility, ectopic pregnancy, and chronic pelvic pain.

Below, we systematically categorize PID animal models by induction method, with technical details and mechanisms summarized in Tables 1, 2, respectively.

**Table 1:** Induction methods for animal models of PID.

Category		Induction method	Animal	Major pathogens/Chemicals/Physical means
Single method	Pathogen-induced	Single pathogen	Female C57BL/6 J mice (31) Female BALB/c mice (31, 42) Female olive baboons (*Papio anubis*) (56) Adult female pig-tailed macaques (*Macaca nemestrina*) (55, 78) British Shorthair domestic cats (60) Female Hartley strain guinea pigs (61)	*Chlamydia muridarum* (31) *Staphylococcus aureus lipoteichoic acid* (42) *Chlamydia trachomatis serotype D* (55) *Chlamydia trachomatis serotype E* (56, 78) *Chlamydia psittaci* (60, 61)
		Multi-pathogen	Female SD rats (28, 32, 35, 75, 76, 98) Female New Zealand White rabbits (30)	*Escherichia coli* and *Staphylococcus aureus* (28) *Escherichia coli* and *Ureaplasma urealyticum* (75, 76) *Escherichia coli* and *Staphylococcus aureus* and *Ureaplasma urealyticum* (35, 98) *Escherichia coli* and *Staphylococcus aureus* and *Beta hemolytic streptococcus* (32) *Escherichia coli* and *Neisseria gonorrhoeae* and *Bacteroides fragilis* and *Peptococcus niger* (30)
	Chemically Induced	Phenol mucilage	Female SD rats (79–84, 86) Female Wistar rats (85)	20% Phenol mucilage (79, 85) 25%Phenol mucilage (82–84, 86) 30% Phenol mucilage (80)
		HCL combined with LPS	Female C57BL/6 J mice (88–90) Female SD rats (99)	HCl and LPS (88–90, 99)
		Estrogen	Female Wistar rats (91)	17-β-estradiol or valerate estradiol (91)
	Physical induction	Foreign body implantation	Female SD rats (92)	Plastic pipe (92)
multi-method combination	Pathogen association with physical induction	Mixed bacterial joint mechanical injury	Female SD rats (17, 63, 69–74, 77)	*Escherichia coli* and *Staphylococcus aureus* + mechanical injury (17, 69–74) *Escherichia coli* and *Staphylococcus aureus* and *Beta hemolytic streptococcus* + mechanical injury (63) *Escherichia coli* and *Staphylococcus aureus* and *Ureaplasma urealyticum* + mechanical injury (77)
	Pathogen combined chemical induction	Mixing bacterial liquid with phenol paste	Female SD rats (33)	*Escherichia coli* and *Staphylococcus aureus* and *Candida albicans* + 7%Phenol mucilage (33)
	Pathogen combined with emergency factors	Pathogens combine fatigue and hunger	Female Wistar rats (29)	*Ureaplasma urealyticum* + fatigue and hunger (29)

**Table 2:** Induction mechanisms for animal models of PID.

Induction category	Specific induction protocol	Core mechanism of action	Key signaling pathways	References
Pathogen-induced	Single pathogen (*Ureaplasma urealyticum*)	*Ureaplasma urealyticum* ascends through the reproductive tract to infect fallopian tube epithelium, activates inflammatory pathways while disrupting CD4^+^/CD8^+^ T cell subset balance, mediates inflammation chronicization, and ultimately drives tissue remodeling and pelvic adhesion	NF-κB pathway, TGF-β fibrosis pathway	(29)
	Single pathogen (*Chlamydia*)	*Chlamydia* forms inclusions in reproductive tract epithelial cells to achieve persistent infection, continuously stimulates the immune system by releasing antigens, triggers massive release of inflammatory factors, and upregulates fibrosis factors to induce fallopian tube adhesion and scar formation	TLR2 pathway, TGF-β fibrosis pathway, miRNA regulatory network (miR-223-3p, miR-155-5p)	(31, 55, 56, 60, 61, 78)
	Multi-pathogen (*Escherichia coli* + *Staphylococcus aureus*)	The two pathogens activate core inflammatory pathways through TLR4 and TLR2 receptors respectively, synergistically trigger inflammatory cascade, and persistent infection activates fibrosis pathway, ultimately inducing chronic sequelae such as pelvic adhesion and decreased endometrial receptivity	TLR4/TLR2-NF-κB pathway, TGF-β/MMPs fibrosis pathway, LIF/JAK2/STAT3 pathway	(17, 28, 69–74)
	Multi-pathogen (*Escherichia coli* + *Ureaplasma urealyticum*)	The two pathogens activate TLR2 and TLR4 receptors, respectively, to achieve synergistic amplification of inflammatory signals, while disrupting inflammatory resolution homeostasis, mimicking the pathological progression of clinical PID from acute to chronic stage	TLR2/TLR4-NF-κB pathway, LXA4/FPR2 inflammatory resolution pathway	(75, 76)
	Multi-pathogen (*Escherichia coli* + *Staphylococcus aureus* + *Beta hemolytic streptococcus*)	The three pathogens synergistically colonize to form biofilms, simultaneously activate NF-κB and MAPK/JNK pathways to form a positive inflammatory feedback loop, driving inflammation chronicization and pelvic fibrosis	NF-κB pathway, MAPK/JNK pathway, TGF-β1/MMP-2 fibrosis pathway, LIF/JAK2/STAT3 pathway	(32, 63, 64)
	Multi-pathogen (*Escherichia coli* + *Staphylococcus aureus* + *Candida albicans*)	Three types of pathogens (Gram-negative bacteria, Gram-positive bacteria, fungi) synergistically activate the TLR4/NF-κB pathway, triggering a vicious cycle of “barrier disruption-persistent infection-inflammation amplification-tissue damage”	TLR4-NF-κB pathway	(33)
	Multi-pathogen (*Escherichia coli* + *Staphylococcus aureus* + *Ureaplasma urealyticum*)	*Ureaplasma urealyticum* first destroys the epithelial barrier, the three pathogens form a mixed biofilm to evade immune clearance, and synergistically activate inflammatory signals through multiple targets, mimicking the pathogenic mechanism of clinical multi-pathogen ascending infection	NF-κB pathway	(77)
	Multi-pathogen (*Escherichia coli* + *Neisseria gonorrhoeae* + *Bacteroides fragilis* + *Peptococcus niger*)	The four pathogens act synergistically in stages: *Neisseria gonorrhoeae* destroys the mucosal barrier, *Escherichia coli* constructs an anaerobic microenvironment, and anaerobic bacteria proliferate massively to mediate inflammatory damage and fibrosis, fully simulating the natural pathogenic pathway of human PID	No separate pathway reported, multi-pathogen synergistically activates inflammatory cascade	(30)
Chemically induced	Phenol mucilage	The corrosive effect of phenol directly causes necrosis and shedding of endometrial epithelium, releases damage-associated molecular patterns to trigger inflammatory cascade, and abnormally activates fibrosis pathway to induce chronic pathological changes such as endometrial hyperplasia and intrauterine adhesion	MAPK/ERK1/2 pathway, TGF-β1/Smad2/3 fibrosis pathway, Th1/Th2 immune imbalance	(33, 79–86)
	HCl combined with LPS	HCl first destroys the endometrial epithelial barrier, allowing LPS to contact deep immune cells, activates the TLR4 pathway to trigger specific pelvic inflammation, and avoids systemic inflammatory response induced by LPS alone	TLR4-NF-κB pathway	(87–90, 99)
	Exogenous estrogen	Long-term estrogen exposure disrupts the immune defense of the reproductive tract mucosa, induces abnormal proliferation of vaginal flora and ascending infection, and spontaneously forms chronic PID manifestations such as tubo-ovarian abscesses and pyometra	Hormone-immune interaction regulatory pathway	(91)
Physically induced	Mechanical injury (used in combination with other induction methods)	Directly destroys the physical barrier of endometrial epithelium, releases damage-associated molecular patterns to synergistically amplify inflammatory signals, and initiates abnormal tissue repair process to induce sequelae such as adhesion and fibrosis	NF-κB pathway, TGF-β/MMP-2 fibrosis pathway, LIF/JAK2/STAT3 pathway	(17, 63, 69–74, 77)
	Foreign body implantation	Acute injury caused by surgical operation + continuous stimulation of implanted foreign body triggers chronic granulomatous inflammation, and simultaneously induces tissue fibrosis and bacterial biofilm formation, mimicking chronic PID associated with intrauterine devices	Foreign body-mediated persistent inflammatory pathway	(92)
Combined induction	Pathogen combined with mechanical injury	Mechanical injury breaks the barrier to improve pathogen colonization efficiency, and damage-associated molecular patterns synergize with pathogen-associated molecular patterns to amplify inflammatory response, improving model stability and pathological fidelity	NF-κB pathway, TGF-β/MMPs fibrosis pathway	(17, 63, 69–74, 77)
	Pathogen combined with low-concentration phenol mucilage	The damage effect of low-concentration phenol mucilage synergizes with pathogen infection to construct a chronic PID model closer to clinical manifestations	NF-κB pathway	(33)
	Pathogen combined with fatigue and hunger stress	Stress reduces host immunity, promotes persistent infection of *Ureaplasma urealyticum*, and mimics the pathological characteristics of clinically recurrent chronic PID	NF-κB pathway, TGF-β fibrosis pathway	(29)

## 3 Pathogen-induced animal models of PID

### 3.1 Pathogens

A diverse array of pathogens is employed in the development of PID animal models. Currently, these pathogens primarily encompass *Escherichia coli*, *Staphylococcus aureus*, *Ureaplasma urealyticum*, *Neisseria gonorrhoeae*, *Bacteroides fragilis*, *Peptococcus niger*, *Chlamydia*, *Hemolytic streptococci*, and *Candida albicans*, among others (28–35). Notably, *Escherichia coli* and *Staphylococcus aureus* are frequently utilized in these models (36). Here, we review the application of these pathogens in the construction of PID animal models.

*Escherichia coli*, a frequently utilized Gram-negative bacterium in PID animal models, induces chronic pelvic diseases primarily through the LPS present on its cell surface (37). LPS, a significant virulence factor, activates the TLR4 signaling pathway within host cells, leading to the release of numerous pro-inflammatory cytokines by inflammatory cells, including TNF-*α* and IL-6 (38, 39). These cytokines can initiate an acute inflammatory response in the pelvic region. If this inflammation is not adequately managed, persistent inflammatory stimulation may result in fibrosis and adhesion of pelvic organ tissues, ultimately progressing to chronic pelvic diseases.

*Staphylococcus aureus*, a representative of Gram-positive bacteria, exhibits a complex mechanism in the induction of PID. The bacterium produces various exotoxins and enzymes, including *α*-hemolysin and coagulase (40), which can directly damage pelvic tissue cells and compromise the integrity of the local mucosal barrier. Additionally, the peptidoglycan and teichoic acid present in the cell wall of *Staphylococcus aureus* can activate the TLR2 signaling pathway (41–43). This activation initiates an immune-inflammatory cascade, resulting in the infiltration of inflammatory cells such as neutrophils and macrophages. Prolonged chronic inflammation can lead to structural and functional alterations in pelvic organs, such as stenosis and obstruction of the fallopian tubes, ultimately culminating in chronic pelvic diseases.

*Ureaplasma urealyticum* is a prokaryotic microorganism characterized by the absence of a cell wall. Its role in inducing PID is primarily associated with its adhesion capabilities and the production of metabolic byproducts (44). This microorganism adheres to the epithelial cells of the genitourinary tract (45), evades the host’s immune defenses, and inflicts direct damage on epithelial cells through the release of toxic metabolic byproducts, such as ammonia and hydrogen peroxide (46). Furthermore, infection with *Ureaplasma urealyticum* can stimulate the host’s production of autoantibodies, thereby triggering an autoimmune response. This response can lead to chronic inflammation and damage to pelvic tissues, ultimately facilitating the onset and progression of chronic pelvic diseases.

*Neisseria gonorrhoeae* is the etiological agent responsible for gonorrhea and is a significant pathogenic bacterium implicated in the development of PID (47). This bacterium primarily adheres to the columnar epithelial cells of the cervical canal via pili, subsequently infiltrating the mucosal epithelial cells and proliferating intracellularly (48). The LPS component of *Neisseria gonorrhoeae* exhibits endotoxin activity, which can activate inflammatory cells to release a substantial amount of inflammatory mediators, resulting in acute suppurative inflammation. Should the infection ascend to the pelvic organs, such as the fallopian tubes and ovaries, it can inflict damage on the fallopian tube mucosa, leading to lumen stenosis or atresia. This can subsequently result in chronic pelvic conditions, including chronic pelvic pain and infertility (49).

*Bacteroides fragilis*, a significant anaerobic Gram-negative bacterium, is integral to the pathogenesis of PID (50–52). The LPS present in its cell wall can activate the TLR4 signaling pathway, thereby inducing an inflammatory response. Furthermore, *Bacteroides fragilis* produces various enzymes, including hyaluronidase and collagenase, which degrade the extracellular matrix of pelvic tissues, facilitating bacterial dissemination and invasion (53). Additionally, this bacterium may modulate the host immune response by inhibiting immune cell function, thereby allowing the infection to persist. This can lead to a chronic inflammatory state, ultimately contributing to the development of chronic pelvic diseases.

*Peptococcus niger* is an anaerobic, Gram-positive coccus. To date, no literature has specifically elucidated the association between this bacterium and PID. However, in the development of animal models, researchers have combined it with other pathogens to successfully induce PID (30). From a microbiological standpoint, it is hypothesized that the mechanism by which *Peptococcus niger* contributes to PID may be linked to its metabolic products and immunomodulatory effects. This bacterium proliferates in anaerobic conditions and can produce metabolic byproducts such as short-chain fatty acids. These substances have the potential to stimulate pelvic tissues and initiate localized inflammatory responses. Additionally, the bacterium may influence host immune cell functions, such as inhibiting macrophage phagocytic activity, thereby complicating the clearance of the infection. Prolonged chronic infection can lead to sustained inflammation and damage to pelvic tissues, potentially progressing to chronic PID.

*Chlamydia trachomatis* is an obligate intracellular pathogen with a distinctive mechanism for inducing PID (54). Researchers utilize *Chlamydia trachomatis*, specifically serotypes D and E, to induce PID models; however, these strains are currently employed exclusively in adult female pig-tailed macaques (*Macaca nemestrina*) (55) and female olive baboons (*Papio anubis*) (56). The pathogen adheres to and invades host cells, forming intracellular inclusions for replication. Upon infection, host cells initiate a range of immune responses, including both cellular and humoral immunity. Nevertheless, *Chlamydia trachomatis* employs various strategies to evade the host’s immune defenses, such as inhibiting apoptosis and disrupting antigen presentation (57), resulting in persistent infection. Women infected with *Chlamydia trachomatis* are at an elevated risk of developing PID (58), including conditions such as salpingitis and endometritis, which can lead to the formation of fallopian tube scarring and obstruction (59), potentially triggering chronic pelvic diseases. In addition, *Chlamydia psittaci* causing guinea pig inclusion body conjunctivitis and feline keratoconjunctivitis are used in animal models of PID, which are typically employed in specific experimental research scenarios (60, 61).

*Hemolytic streptococci* constitute a group of bacteria capable of producing hemolysins, which are implicated in the pathogenesis of PID primarily through their toxic effects and the consequent inflammatory response (62). *Beta hemolytic streptococcus*, also known as *β-hemolytic streptococci*, are frequently studied in this context (32, 63, 64). Hemolysins exert their pathogenic effects by lysing red blood cells and damaging other tissue cells, leading to hemorrhage and tissue damage within the pelvic region. Concurrently, components of the *hemolytic streptococci* cell wall can activate the host immune system, eliciting a robust inflammatory response (65). This response is characterized by the infiltration of a substantial number of inflammatory cells into the pelvic tissues. Persistent inflammation may result in structural damage and functional impairment of pelvic organs, ultimately progressing to PID.

*Candida albicans* is a prevalent opportunistic pathogenic fungus, with its pathogenicity primarily associated with hyphal formation and immune evasion (66). A study entitled “Pelvic Inflammatory Disease in the People’s Republic of China: Aetiology and Management” reported that the prevalence of *Candida albicans* in patients with PID was 11% (67). Under favorable conditions, *Candida albicans* can transition from the yeast phase to the hyphal phase (66), thereby enhancing its adhesion and invasion capabilities in pelvic tissues. The hyphae are capable of penetrating mucosal epithelial cells, resulting in tissue damage. Furthermore, *Candida albicans* can modulate the host’s immune response by inhibiting the chemotaxis and phagocytic functions of neutrophils (68), which facilitates persistent infection and contributes to chronic pelvic inflammation.

### 3.2 Methods and mechanisms

The single pathogen-induced model involves simulating the occurrence of PID through the inoculation of a singular pathogen. Although this approach is not widely employed in the study of PID pathogenesis, it retains some reference value. Previous studies have identified the use of specific pathogens in this model, including *Ureaplasma urealyticum* (29), *Chlamydia muridarum* (31), *Chlamydia trachomatis serotype E strain* (56), and *Chlamydia trachomatis serotype D strain* (55).

Mixed infection with multiple pathogens involves the simultaneous introduction of two or more pathogens into the same animal model to replicate complex clinical infection scenarios. This approach more accurately mirrors actual clinical conditions and is frequently employed to induce infections, thereby providing a more comprehensive understanding of the synergistic effects of multiple pathogens on the host’s immune response and pathological alterations. Currently, documented combinations of multiple pathogens include *Escherichia coli* and *Staphylococcus aureus* (17, 28, 69–74); *Escherichia coli* and *Ureaplasma urealyticum* (75, 76); *Escherichia coli*, *Staphylococcus aureus*, and *Beta hemolytic streptococcus* (32, 63, 64); *Escherichia coli*, *Staphylococcus aureus*, and *Candida albicans* (33); *Escherichia coli*, *Staphylococcus aureus*, and *Ureaplasma urealyticum* (77); and *Escherichia coli*, *Neisseria gonorrhoeae*, *Bacteroides fragilis*, and *Peptococcus niger* (30). These composite infection models are inoculated through different routes, such as intrauterine injection, vaginal perfusion, or combined surgical intervention, to induce a more complex PID reaction.

#### 3.2.1 Ureaplasma urealyticum

In the experimental procedure involving the induction of a singular pathogen, *Ureaplasma urealyticum*, researchers administered vaginal injections of *Ureaplasma urealyticum* to 12-week-old unmated female Wistar rats (weighing 220 ± 20 g). The specific protocol was as follows (29): After a one-week acclimatization period, the rats received vaginal injections of a liquid culture of *Ureaplasma urealyticum* on the 12th, 13th, and 14th days, with each injection consisting of 0.5 mL. On the 25th day, the rats were euthanized, and their uterine and fallopian tube tissues were collected for pathological examination. It is important to note that this method involves the selection of a single type of pathogen; however, studies often incorporate non-specific stressors such as fatigue and hunger to increase infection success rate.

Against a backdrop of impaired immunity caused by fatigue and hunger, *Ureaplasma urealyticum* ascends through the reproductive tract to infect the fallopian tube epithelium. By activating the NF-κB signaling pathway, it releases pro-inflammatory cytokines such as TNF-*α*, IL-6, and IL-17, which chemotactically attract immune cells to infiltrate the site, leading to mucosal edema, epithelial necrosis, and luminal obstruction; simultaneously disrupting the balance of CD4^+^/CD8^+^ T-cell subsets. Although Th2 cytokines IL-4 and IL-10 are compensatorily elevated, they fail to reverse the immune dysregulation, thereby mediating the chronicization of inflammation. Subsequently, highly expressed TGF-*β* induces fibroblast activation; in conjunction with upregulated MMP-2, which degrades the basement membrane, and VEGF-β, which promotes angiogenesis, these factors collectively drive tissue remodeling and pelvic adhesions, ultimately resulting in the characteristic pathological changes of chronic pelvic inflammatory disease (CPID) (29).

#### 3.2.2 Chlamydia

The single pathogen induction model for *Chlamydia* employs both *Chlamydia trachomatis* and *Chlamydia muridarum* to induce genital tract infections (31), thereby providing a viable method for *Chlamydia muridarum* to simulate human *Chlamydia trachomatis* infections. Currently, the induction of clinical isolates of *Chlamydia trachomatis serotype E* has been applied exclusively in *Papio anubis* and *Macaca nemestrina* (56, 78). The procedures are as follows (56): The isolate is directly instilled onto the external cervix of *Papio anubis* using a pipette once a week for a total of five inoculations, each consisting of 1 × 10^7^ inclusion-forming units. The animals are euthanized 16 weeks following the initial inoculation, at which point final samples are collected. Alternatively, *Macaca nemestrina* is inoculated through cervical flushing (78) with a *Chlamydia trachomatis* strain of serotype E at a concentration of 5 × 10^4^ infectious units (IFU) per milliliter, administered once a week for a total of five inoculations. At present, the induction of the *Chlamydia trachomatis serotype D* strain has been exclusively documented in *Macaca nemestrina* (55). Each inoculum comprises 5.9 × 10^9^ inclusion-forming units (IFU) per milliliter, prepared in a sucrose-phosphate-glutamate buffer. The inoculation is administered directly through the fimbrial opening, with 0.15 mL of the inoculum introduced into each fallopian tube. The monkeys undergo three inoculations, spaced 2 weeks apart. A hysterectomy is conducted 12–16 weeks following the final inoculation, resulting in a total study duration of approximately 22 weeks. Beside, *Chlamydia psittaci* has been used to induce PID in British Shorthair cats and Hartley guinea pigs (60, 61). In cats (60), laparoscopic injection of 0.1 mL inoculum (100 × 10^9^ IFU/L) into fallopian tube fimbriae resulted in acute (4-18 day), chronic (≈30 day), and scarring/adhesion (40-50d) stages. Guinea pigs (61) received 10^6^–10^7^ IFU via vaginal inoculation and were evaluated pathologically 30 day post-infection.

The microscopic mechanism underlying *Chlamydia trachomatis*-induced PID is as follows: after the pathogen ascends through the cervix and invades the upper reproductive tract, it causes irreversible damage to the fallopian tube tissue by persisting and abnormally activating the host’s immune inflammatory pathways. In macaque, baboon, and mouse models, repeated *Chlamydia* inoculation allows the pathogen to stably colonize the cervix and spread to the fallopian tube epithelium (31, 78). On the one hand, it evades clearance by forming inclusions, maintaining a low-level persistent infection, and continuously stimulating the host by releasing antigens such as CHSP60 and major outer membrane protein; on the other hand, it triggers the massive release of early pro-inflammatory factors (TNF-*α*, IL-1*β*, CXCL1/2, IL-8) via the TLR2 pathway, recruiting plasma cells, neutrophils, and lymphocytes to form severe inflammatory infiltration, while simultaneously upregulating the key fibrogenic factor TGF-β, leading to tubal adhesions, edema, dilation, and scar formation; Furthermore, differential expression of miRNAs (such as miR-223-3p and miR-155-5p) in the early stages of infection can negatively regulate inflammatory signaling, serving as molecular biomarkers for predicting severe PID outcomes such as hydrosalpinx (31, 78). Together, these factors constitute the core molecular mechanism of the PID model: “persistent Chlamydia infection—immune hyperactivation—inflammation-fibrosis cascade—structural damage to the upper reproductive tract.”

#### 3.2.3 *Escherichia coli* and *Staphylococcus aureus*

The co-administration of *Escherichia coli* and *Staphylococcus aureus* is the most extensively utilized method (17, 28, 69–74). Currently, this combination of bacterial solutions is frequently employed in the development of the PID model in female Sprague–Dawley (SD) rats (17, 28, 69–74). The ratio of *Escherichia coli* to *Staphylococcus aureus* is typically either 1: 1 or 2:1. The concentration of the bacterial solution generally ranges from 10^8^ to 10^9^ CFU/mL, with an inoculation volume of 0.1 to 0.2 mL per administration. This solution is introduced into the uterus or vagina via abdominal surgery or vaginal injection (17, 28, 69–74). Initially, the surgical procedure involves anesthetizing the rats (17, 69, 71, 73, 74), followed by performing an abdominal incision under aseptic conditions. The incision is strategically positioned near the intersection of the line connecting the two knee joints and the mid-abdominal line when the rat is in the supine position. The skin and abdominal wall muscles are incised to expose the uterine horns, subsequently revealing the “Y”-shaped uterus. A mixed bacterial solution is then carefully administered into the left and right uterine horns using a microsyringe, with the needle inserted at the uterine bifurcation to ensure uniform distribution of the solution. Upon completion of the procedure, the incision is sutured, and anti-infection care is provided. Typically, a single operation suffices, with the modeling duration ranging from 10 to 14 days. However, in certain studies, this period may vary from as short as 3 days or 24 h to as long as 60 days. Various methods are employed for model evaluation, including uterine anatomical morphological scoring, physiological indicators, bacteriological detection, histopathological examination, and blood routine examination, among others (17, 69, 71, 73, 74). These methods can be used to evaluate the degree of inflammation, tissue damage, and changes in the immune response caused by infection, and to determine whether the model is successfully established. Secondly, the non-surgical approach involves vaginal perfusion inoculation, which can be primarily categorized into bacterial solution injection and small gelatin sponge implantation. Bacterial solution injection entails administering the bacterial solution into the uterine cavity via a syringe equipped with a blunted needle tip, entering through the uterine cervical bifurcation at the base of the vagina (70, 72). The concentration and volume of the bacterial solution, as well as the frequency of operations and the duration of the modeling process, are consistent with those employed in the aforementioned surgical procedure (70, 72). In the gelatin sponge implantation method, a small gelatin sponge saturated with the bacterial solution is inserted into the rat’s cervix, and the rat is positioned upside down for 2 to 3 min. This procedure is repeated every other day for a total of four sessions, culminating in a total modeling period of 12 days (28).

The microscopic pathway mechanism underlying the PID induced by *Escherichia coli* and *Staphylococcus aureus* involves these two pathogens activating the TLR4 and TLR2 receptors, respectively, through their characteristic pathogen-associated molecular patterns (28, 72), thereby synergistically triggering the NF-κB core inflammatory pathway (28, 72, 73): LPS from the Gram-negative bacterium *E. coli* is recognized by TLR4, while peptidoglycan and lipoteichoic acid from the Gram-positive bacterium *Staphylococcus aureus* are recognized by TLR2. Both activate IKK via the MyD88-dependent pathway, leading to the phosphorylation and degradation of IκB and promoting the phosphorylation and nuclear translocation of the NF-κB p65 subunit, transcribing and releasing pro-inflammatory factors such as IL-1β, TNF-*α*, and IFN-*γ*, as well as NO, which recruit inflammatory cells—including neutrophils and lymphocytes—to infiltrate the endometrial mucosa and myometrium, resulting in acute epithelial necrosis, intrauterine congestion and edema, and damage to the ultrastructure of mitochondria and microvilli; Persistent pathogen infection and inflammation further activate the TGF-*β*/MMPs core fibrosis pathway (17, 73), promoting collagen synthesis and excessive ECM deposition by upregulating the pro-fibrotic factor TGF-β1, while simultaneously downregulating MMP-2 expression to inhibit ECM degradation. Combined with inhibition of the LIF/JAK2/STAT3 pathway and sustained activation of oxidative stress, this ultimately leads to chronic sequelae of PID, such as intrauterine/pelvic adhesions, reduced endometrial receptivity, and other chronic sequelae of PID.

#### 3.2.4 *Escherichia coli* and *Ureaplasma urealyticum*

The concurrent inoculation of *Escherichia coli* and *Ureaplasma urealyticum* has thus far been documented exclusively by the research team led by *Zou W*. This methodology was employed in two successive studies (75, 76). Female SD rats were utilized as the experimental subjects. The concentration of *Escherichia coli* and *Ureaplasma urealyticum* was maintained at 1 × 10^8^ CFU/mL, although the specific volume was not detailed. In each instance, a gelatin sponge with a volume of 0.125 mL, saturated with a mixture of the aforementioned pathogens, was utilized. This bacteria-laden gelatin sponge was subsequently implanted into the cervix of each rat in the PID group, with the rats being positioned upside-down for a duration of 3 min (76). In contrast, the control group received gelatin sponges soaked in normal saline (76). This infection procedure was conducted bi-daily and was repeated a total of four times (76). In subsequent experiments, to ensure control over variables, the rats underwent pre-treatment with hormones. Following a 7-day acclimatization period, all rats received a subcutaneous injection of progesterone (10 mg) for 7 days to synchronize their estrous cycles, as outlined in reference [75]. The previously established method was then employed for model establishment, as detailed in reference [76]. Depending on the specific experimental objectives, the duration of model-building trials varied, ranging from as brief as 24 h (75) to as extended as 21 days (76). As previously described, model evaluation methods, including uterine morphological observation, histopathological examination, bacterial culture, and the detection of inflammatory factors, were utilized to assess the degree of infection in the model and the status of the immune response (75, 76).

The combined use of *Escherichia coli* and *Ureaplasma urealyticum* to establish a PID rat model essentially involves the two pathogens synergistically triggering an inflammatory response in the upper reproductive tract of the host via pattern recognition receptors, thereby disrupting the endogenous inflammatory resolution homeostasis and simulating the acute and chronic pathological processes of multi-pathogen infections observed in clinical PID (75, 76). The lipid-associated membrane protein of *Ureaplasma urealyticum* is recognized by TLR2, while the lipopolysaccharide of *Escherichia coli* is recognized by TLR4; both activate intracellular inflammatory signaling through MyD88-dependent pathways. Compared with single-pathogen infection, combined stimulation by two pathogens activates two TLR subtypes, creating a synergistic amplification of signaling that triggers an innate immune response in the upper genital tract; this constitutes the pathogenetic basis for the combined model’s replication of the pathological features of clinical PID. Activated TLR2/4 activates the IKK complex via the MyD88 adaptor molecule, mediating the degradation of IκBα, which allows NF-κB p65 to translocate to the nucleus and activate pro-inflammatory cytokines and chemokines. These inflammatory mediators lead to damage to upper genital tract epithelial cells, stromal edema, and the chemotaxis and infiltration of immune cells, resulting in the histopathological changes characteristic of PID. Concurrently, the dual pathogens excessively activate the TLR/NF-κB signaling pathway, disrupting the homeostasis of inflammatory resolution at both the synthetic and effector levels. This occurs through two mechanisms: first, by depleting arachidonic acid, leading to insufficient LXA4 synthesis; and second, by inhibiting FPR2 expression, thereby weakening the effects of LXA4. Consequently, inflammation fails to resolve, mimicking the pathological progression of clinical PID from acute to chronic stages.

#### 3.2.5 *Escherichia coli, Staphylococcus aureus,* and *Beta hemolytic streptococcus*

The study on the PID model involving the combined inoculation of *Escherichia coli*, *Staphylococcus aureus*, and *Beta hemolytic streptococcus* employs either surgical techniques or vaginal inoculation. Female Wistar or SD rats are utilized as the experimental subjects (32, 64). The bacterial mixture is prepared in a ratio of 2:1:1 for *Escherichia coli*, *Staphylococcus aureus*, and *Beta hemolytic streptococcus*, respectively (32, 64). Notably, only one study documented a total bacterial concentration of 6 × 10^10^ CFU/mL. The surgical inoculation procedure aligns with previously described methodologies (17, 69, 71, 73, 74), wherein the uterine horns are accessed via laparotomy, and a mixed bacterial solution is administered. The injection sites may vary, including bilateral injections or unilateral injections. To prevent leakage of the bacterial solution, a gelatin sponge measuring 0.5 cm × 0.5 cm may be inserted into the vagina prior to the procedure (63). For vaginal inoculation, two gelatin sponges, each measuring 0.5 cm × 0.5 cm and saturated with the bacterial mixture, are introduced into the uterine cavity through the vaginal route (32). The model-building time is 7 days or 14 days. There is no difference in the model evaluation methods.

*Escherichia coli*, *Staphylococcus aureus*, and *Beta-hemolytic Streptococcus* jointly establish a rat PID model, revealing the core mechanisms of the microscopic pathways. This process represents a linear regulatory cascade involving multi-pathogen synergistic invasion, pattern recognition, amplification of inflammatory signaling, and progression of pathological phenotypes (64): the three bacteria synergistically adhere and invade via fimbriae/LPS, surface proteins/coagulase, and hemolysin/M protein, respectively, forming biofilms to achieve sustained colonization (64, 69, 73, 74); Their pathogen-associated molecular patterns are recognized by TLR4 and TLR2, triggering a MyD88-dependent signaling cascade. This activates the IKK complex, mediating the phosphorylation and degradation of IκB*α*, driving the nuclear translocation of NF-κB p65 (64, 73, 74), while simultaneously synergistically activating the MAPK/JNK pathway to form an inflammatory positive feedback loop, resulting in the massive transcription and release of pro-inflammatory factors such as IL-1β, IL-6, and TNF-α, which disrupt the immune homeostasis of pro-inflammatory and anti-inflammatory cytokines and mediate the massive chemotaxis and infiltration of inflammatory cells (64, 73, 74); Persistently activated NF-κB, in conjunction with abnormally high levels of TGF-β1, on the one hand inhibits the p53/Fas/FasL pathway and disrupts the Bax/Bcl-2 balance, leading to a block in inflammatory cell apoptosis and creating a vicious cycle of continuously amplified inflammation (64); on the other hand, it disrupts the TGF-β1/MMP-2 pathway, driving abnormal extracellular matrix deposition and pelvic fibrosis and adhesions (64, 73); Concurrently, inflammatory signals suppress the endometrial LIF/JAK2/STAT3 pathway, disrupting the homeostasis of the gut microbiota and amplifying inflammatory damage via the gut-reproductive axis. This ultimately leads to the progression of acute inflammation into chronic PID, fully replicating the pathological progression of clinical PID and the sequelae of reproductive dysfunction (64, 69, 73).

#### 3.2.6 *Escherichia coli, Staphylococcus aureus,* and *Candida albicans*

A PID model with triple inoculation of *Escherichia coli*, *Staphylococcus aureus*, and *Candida albicans* (33). The concentration of the mixed bacterial solution was 6.0 × 10^8^ CFU/mL. Vaginal injection was used, with 0.15 mL injected each time, for a total of 3 times. However, before model establishment, the researchers performed ovariectomy on the rats to eliminate the influence of endogenous hormone fluctuations on the infection process (33).

Three types of pathogens—Gram-negative bacteria, Gram-positive bacteria, and fungi—work synergistically to activate the host’s TLR4/NF-κB inflammatory signaling pathway through their distinct pathogen-associated molecular patterns (33). This triggers a cascade of pro-inflammatory cytokine release, massive infiltration of inflammatory cells, and oxidative stress-induced damage, creating a vicious cycle of “barrier disruption—persistent infection-inflammation amplification-tissue damage,” ultimately establishing a rat model with pathological features highly consistent with clinical PID.

#### 3.2.7 *Escherichia coli, Staphylococcus aureus,* and *Ureaplasma urealyticum*

Mixed inoculation of *Escherichia coli*, *Staphylococcus aureus*, and *Ureaplasma urealyticum* (77). 0.3 mL of the mixed bacteria solution (2 × 10^12^ cells/L; diluted at a ratio of 1: 1:1) was slowly injected into the uterine cavity of female SD rats through the cervix (77). There was no significant difference from the above—mentioned operation.

A rat PID model was established using a combination of *Escherichia coli*, *Staphylococcus aureus*, and *Ureaplasma urealyticum*. By leveraging the complementary virulence characteristics and pathogenic phenotypes of these three clinically predominant PID pathogens, the model replicates the pathogenic mechanism of clinical multi-pathogen ascending infections, thereby creating a stable, standardized disease model that aligns with clinical pathological features (77). *Ureaplasma urealyticum* first invades damaged epithelium (Due to their lack of a cell wall and high adhesive capacity, these pathogens preferentially invade damaged epithelial tissue), disrupting the genital tract microenvironment and compromising epithelial cell membrane integrity, thereby creating conditions for the adhesion and colonization of the other two bacteria. The three pathogens form a mixed biofilm to evade immune clearance, enabling persistent infection of the upper genital tract. After colonization, the three bacteria form a multi-target synergistic activation effect through their respective pathogen-associated molecular patterns, activating multiple pathways to amplify downstream NF-κB inflammatory signals, driving the transcription and translation of pro-inflammatory factors, and forming an inflammatory feedback loop. Ultimately, excessive inflammatory factors recruit and infiltrate peripheral immune cells into the endometrium, activate matrix metalloproteinases to disrupt glandular and stromal structures, and induce epithelial cell necrosis and apoptosis, resulting in a pathological phenotype consistent with clinical acute PID. Furthermore, the persistent infection by the three bacteria and the sustained activation of inflammatory signals ensure the stability of the experimental window for drug intervention following model establishment.

#### 3.2.8 *Escherichia coli, Neisseria gonorrhoeae, Bacteroides fragilis,* and *Peptococcus niger*

Regarding the combined infection of *Escherichia coli*, *Neisseria gonorrhoeae*, *Bacteroides fragilis*, and *Peptococcus niger* (30), the researchers used a mixed bacterial solution (the mixing ratio was not specified) and injected it into the uterine cavity of Pasteurella-free New Zealand white rabbits (1-year-old, weighing 2.8–3.0 kg) through vaginal-cervical intubation. Specific operation: An intrauterine artificial insemination catheter (5.3F Soules IUI catheter) was inserted through the cervix to the 7-cm mark, and 5 mL of the bacterial suspension (a total inoculum of 5 × 10^7^ CFU per strain per rabbit) was injected within 5 min (30). The model was established for 7 days. The model was evaluated by laparoscopic digital imaging and histopathological analysis.

This study established a four-bacterial-species PID model consisting of *Escherichia coli*, *Neisseria gonorrhoeae*, *Bacteroides fragilis*, and *Peptococcus niger*, to precisely recapitulate the natural ascending aerobic-anaerobic mixed infection pathway of human PID. The complete pathological process is driven by the stepwise synergistic pathogenicity of the four strains: *Neisseria gonorrhoeae* initiates infection by invading the reproductive tract epithelium via adhesins, releasing lipopolysaccharide to disrupt the mucosal barrier and facilitate bacterial ascending migration; facultatively anaerobic *Escherichia coli* subsequently adheres to the damaged epithelium to expand barrier defects, and creates an anaerobic microenvironment via oxygen consumption during aerobic metabolism, acting as the core hub for aerobic-anaerobic bacterial synergism; *Bacteroides fragilis* and *Peptococcus niger* proliferate vigorously in the anaerobic microenvironment, with the former mediating immune evasion and pro-inflammatory/pro-fibrotic factor release, and the latter forming symbiotic biofilms with *Bacteroides fragilis* to resist immune clearance, which jointly aggravate tubal and peritoneal inflammatory injury. Ultimately, the four strains synergistically induce tubal adhesion/obstruction and abnormal pelvic fibrin deposition, fully recapitulating the core pathological progression of human PID from acute infection to chronic adhesion and infertility, and providing a clinically relevant model for evaluating PID intervention strategies.

## 4 Methods for chemically inducing PID

Chemical induction represents a significant approach for simulating PID, primarily by eliciting an inflammatory response through the administration of chemical agents into animal models. In comparison to the pathogen induction method, chemical induction offers advantages such as ease of operation, reduced modeling time, and high reproducibility. Presently, widely utilized chemical inducers encompass phenol mucilage, hydrochloric acid (HCl) in conjunction with lipopolysaccharides (LPS), and estrogen.

### 4.1 Methods and mechanisms

#### 4.1.1 Phenol mucilage

Phenol mucilage is widely utilized as a chemical inducer in research (33, 79–86). The primary constituent, phenol, is known for its potent irritant and corrosive properties, which can directly harm tissue cells and elicit localized inflammatory responses. In the development of animal models for PID, phenol mucilage is typically administered into the uterus or vagina of animals to replicate the clinical progression of PID. Various methods exist for preparing phenol mucilage, including combinations such as phenol with sodium carboxymethyl cellulose mucilage (79), and phenol with tragacanth, glycerol, and distilled water (82). The concentrations of phenol mucilage employed can differ, including 7, 20, 25, and 30% (33, 79, 80, 84). Despite the variations in composition and concentration, and the absence of a standardized protocol, the fundamental mechanism remains the induction of tissue congestion, edema, and inflammatory cell infiltration via the local stimulatory effects of phenol. Additionally, this approach aligns well with the requirements of traditional Chinese medicine (TCM) syndrome research. Simulating the local pelvic environment of dampness and blood stasis facilitates the observation of the intervention effects of TCM herbs with the functions of promoting blood circulation to remove stasis and clearing heat and promoting diuresis.

The induction of a PID model using phenol mucilage is typically conducted on female rats (SD or Wistar strains) (33, 79–86). The administered dosage ranges from 0.06 mL to 0.1 mL, with a concentration predominantly at 25% (33, 79–86). When inducing PID with pure phenol mucilage, without the incorporation of pathogens, a standardized abdominal surgical procedure is employed (79–86). The injection is typically administered into one uterine horn, with the contralateral horn serving as a self-control. The modeling duration generally spans 7 to 15 days, with a 1-day duration being exceptionally rare (83). Evaluation methods for this model align with those utilized in pathogen-induced models and include visual assessment of uterine pathological alterations, calculation of uterine swelling and inhibition rates, measurement of serum inflammatory markers, hemorheological analysis, and histopathological examination of uterine tissues, among others (33, 79–86).

The microscopic mechanism underlying the phenol mucilage-induced rat PID model begins with chemical tissue damage as the initial trigger. This initiates a cascade of signaling pathways that drives the complete pathological process, including the onset of acute inflammation, the progression of chronic inflammation, and fibrotic remodeling. The core molecular and cellular events form a tightly regulated network: Upon injection of phenol mucilage into the rat uterine cavity, its corrosive chemical action first causes degeneration, necrosis, and sloughing of endometrial epithelial cells, disrupting the integrity of the uterine mucosal barrier. Simultaneously, this releases large amounts of damage-associated molecular patterns (DAMPs), activating resident innate immune cells such as macrophages and neutrophils within the uterine cavity, thereby initiating the inflammatory response (84, 86); In both the damaged epithelial cells and the activated immune cells, the ERK1/2 pathway of the MAPK family is abnormally activated, with its phosphorylation levels significantly upregulated. This subsequently drives the high expression of downstream pro-inflammatory mediators iNOS and COX-2 at both the transcriptional and protein levels. Among these, iNOS continuously catalyzes the production of excessive NO, while COX-2 mediates the massive synthesis of PGE2. Together, these factors exacerbate local vasodilation and increased vascular permeability, leading to endometrial congestion and edema (84), while simultaneously further promoting the chemotaxis and activation of immune cells, resulting in the initial amplification of inflammatory signals; As inflammatory signaling continues to be activated, activated immune cells secrete large amounts of upstream pro-inflammatory cytokines such as TNF-*α* and IL-1β. Among these, TNF-α further stimulates vascular endothelial cells to express adhesion molecules, recruiting peripheral blood lymphocytes, monocytes, and plasma cells to infiltrate the endometrium, myometrium, and even the serosal layer in large numbers. Concurrently, this triggers a cascade of events leading to the release of downstream inflammatory factors and chemokines such as IL-6 and IL-8, and other downstream inflammatory and chemotactic factors. As a potent chemotactic factor, IL-8 continuously amplifies local leukocyte infiltration and inflammatory activation (79, 84, 86). IL-6, in turn, drives systemic inflammatory responses and local tissue damage. The excessive secretion of pro-inflammatory factors simultaneously suppresses the expression of the Th2 anti-inflammatory factor IL-4, disrupting the Th1/Th2 immune balance. This prevents the inflammatory response from being normally downregulated, promoting the transition of inflammation from an acute to a chronic, persistent state (86); In a persistent chronic inflammatory microenvironment, the TGF-β1/Smad2/3 fibrosis pathway is abnormally activated. Inflammatory cells and activated fibroblasts highly express TGF-β1, which, upon binding to cell membrane receptors, induces the phosphorylation of Smad2/3 proteins; Upon nuclear translocation, phosphorylated Smad2/3 initiates the transcription of fibrosis-related genes. On one hand, this significantly upregulates tissue TIMP-1 expression, inhibiting the degrading effects of MMP-2 and MMP-9 on the extracellular matrix (ECM) and disrupting the physiological balance between ECM synthesis and degradation; on the other hand, directly driving the proliferation and activation of fibroblasts and the massive synthesis of collagen fibers, ultimately leading to endometrial fibrotic hyperplasia, intrauterine adhesions, and obstruction of the fallopian tube lumen (84). This model fully replicates the core sequelae—such as chronic pelvic pain and infertility—resulting from the protracted course of PID, establishing a complete pathological pathway of “chemical injury–acute inflammatory cascade-immune imbalance-chronic fibrotic remodeling,” which is also the core mechanism by which this model can stably simulate the pathological characteristics of the entire course of PID (79, 84, 86).

### 4.2 HCl combined with LPS

The combination of HCl and LPS constitutes a chemical approach for inducing the PID model. HCl is known to inflict chemical damage on tissues, whereas LPS, a principal component of bacterial endotoxins, exerts a potent immunostimulatory effect. It activates immune cells within the organism, leading to the release of inflammatory mediators and thereby intensifying the inflammatory response (87). The PID model induced by the combined application of HCl and LPS is an experimental paradigm established through composite stimulation. Typically, the uterine or cervical mucosa of animal subjects is pre-treated with an HCl solution to compromise the local tissue barrier and induce chemical damage, thereby initiating a more pronounced inflammatory cascade (87).

The modeling method utilizing HCl in conjunction with LPS predominantly involves female C57BL/6 J mice (88–90), although there are also instances of employing female SD rats (91). The concentration of HCl used is 1 N, with a dosage of 25 mg/kg, while LPS is typically administered at a dosage of 50 mg/kg. The duration of the modeling process is generally maintained within a 24-h period. The preferred administration route for this modeling technique is vaginal injection, wherein the subjects receive a single intracervical injection of HCl (25 mg/kg, 1 N), followed by LPS (25 mg/kg or 50 mg/kg) administered every 2 h for a total of four doses (87, 89). Notably, alternative methods such as intraperitoneal injection or laparotomy for uterine perfusion were deliberately avoided to minimize systemic reactions (87, 89). Conversely, another study adopted a different approach, utilizing laparotomy for uterine perfusion and intraperitoneal injection to establish the model (88). In this instance, after anesthetizing the mice, a midline abdominal incision was made to expose the uterus, into which a needle was longitudinally inserted for the injection of HCl (1 N). Subsequently, starting 2 h post-HCl injection, LPS (50 mg/kg) was administered intraperitoneally every 2 h for a total of four doses. Both methods effectively elicit an acute inflammatory response in uterine tissue, characterized by cervical congestion, uterine wall thickening, and infiltration of inflammatory cells. Compared to the first method, laparotomy for uterine perfusion with HCl combined with intraperitoneal injection of LPS allows for more precise localization of HCl action, thereby minimizing the spread of irritation that may occur with vaginal administration. Furthermore, intraperitoneal injection of LPS maintains a relatively high systemic concentration of the drug, enhancing the synchrony of the systemic inflammatory response, which is advantageous for investigating the effects of systemic inflammation on pelvic tissues. Additionally, in comparison to animal models of endometritis induced solely by LPS, this combined approach more closely resembles the clinical progression of PID. The model evaluation methods, excluding pain behavior tests such as the acetic acid writhing test and hot-plate test, are generally consistent, with no significant differences observed.

The microscopic mechanism underlying the establishment of a mouse PID model via sequential cervical administration of HCl combined with LPS lies in the precise sequential synergy between the epithelial barrier-disrupting effect of hydrochloric acid and the innate immune-activating effect of lipopolysaccharide (87). The complete molecular pathway and cascade of reactions begin with a targeted breach of the physiological defense system of the non-pregnant uterus: Following a single transvaginal administration of 25 mg/kg 1 N HCl, the solution directly damages endometrial epithelial cells and disrupts the tight junctions between epithelial cells, significantly increasing the permeability of the genital tract mucosa. Simultaneously inducing mild necrosis and degeneration of the epithelial layer and pre-activating resident macrophages (90). This completely disrupts the high resistance to infection and immune homeostasis inherent in the non-pregnant uterus, creating the critical histological and immunological prerequisites for the subsequent biological effects of LPS. This is also the core reason why LPS administration alone cannot reliably induce PID. An intact uterine epithelial barrier effectively prevents LPS from coming into contact with endometrial stromal cells and deep-seated immune cells, whereas intraperitoneal LPS administration triggers lethal sepsis due to widespread activation of TLR4 in systemic tissues, failing to produce a specific inflammation confined to the pelvic reproductive organs. Once this barrier is compromised, four doses of 50 mg/kg of *Salmonella enterica*-derived LPS administered transvaginally at 2-h intervals can successfully penetrate the damaged epithelial barrier. The LPS then directly binds to TLR4 on the surface of endometrial epithelial cells, stromal cells, uterine glandular cells, and resident macrophages, thereby recruiting MyD88 as an adaptor protein to activate downstream TRAF, initiating the phosphorylation cascade of the NF-κB signaling pathway, promoting the nuclear translocation of the NF-κB p65 subunit, and initiating the transcription and translation of the three core pro-inflammatory cytokines: IL-1β, IL-6, and TNF-*α*; These pro-inflammatory cytokines, on the one hand, further activate the NF-κB pathway through a positive feedback loop, amplifying local pelvic inflammatory signals; on the other hand, they significantly upregulate the expression of ICAM-1 and VEGF, mediating the massive chemotaxis, transendothelial migration, and *in situ* infiltration of neutrophils from the bloodstream into the endometrium and the epithelial layer of the uterine cavity (87, 90). This ultimately results in a microscopic phenotype that closely corresponds to the pathological features of clinical PID: massive leukocyte infiltration (predominantly neutrophils) in the endometrium and epithelium, epithelial cell necrosis and degeneration, intrauterine congestion, hemorrhage, and purulent exudation. Furthermore, because the route of administration is local to the cervix, the inflammatory response is strictly confined to the pelvic reproductive organs, thereby avoiding the severe systemic adverse reactions caused by systemic exposure to LPS and enabling the stable and reproducible establishment of an animal model of PID.

### 4.3 Estrogen

Estrogen has been shown to compromise the innate immune defense of the reproductive tract mucosa, facilitate the overgrowth of vaginal flora, and lead to ascending infections. Consequently, researchers conducted a study in which they developed a spontaneous PID rat model through the exogenous administration of estrogen (91). Adult female Wistar rats, weighing between 110 and 130 grams, were selected for the experiment and divided into three groups. Two groups received continuous estrogen treatment via subcutaneous implantation of 50 mg 17-*β* estradiol slow-release pellets or weekly injections of estradiol valerate (0.166 mg/kg). The third group additionally received amoxicillin treatment starting on the 90th day of hormone exposure. All estrogen-treated rats began to exhibit weight loss after approximately 3 months, with some animals dying spontaneously. Necropsy identified abscesses in the ovaries, fallopian tubes, and uterus. Surviving animals were euthanized at 6 months, and pathological examination confirmed that most developed tubo-ovarian abscesses or pyometra. No significant inflammatory lesions were observed in the 30 control rats. The six-month model establishment period effectively recapitulates the pathological progression of chronic PID. A notable advantage of this model is its ability to induce inflammation spontaneously through alterations in the endocrine environment, without necessitating exogenous pathogen inoculation or surgical intervention. This feature renders the model more representative of the pathological characteristics observed in certain clinical patients who exhibit hormonal disorders without a clear history of infection. Furthermore, the structural damage to the reproductive tract and the chronic inflammatory response induced by prolonged estrogen exposure closely replicate the histological changes associated with human PID. This provides an optimal platform for investigating hormone-immune interactions and the mechanisms underlying chronic infections. Nonetheless, the extended duration required for model establishment, significant individual variability, and the absence of a dynamic observation window for the acute phase limit its utility in the rapid screening of pharmaceutical agents.

Long-term exposure to exogenous estrogen disrupts the innate and adaptive immune responses of the reproductive tract mucosa in non-spayed female rats, inhibiting the secretion of local bactericidal factors, reducing antigen presentation and antibody secretion, and simultaneously inducing keratinization and hyperplasia of the vaginal and cervical epithelium. This facilitates bacterial adhesion and colonization, leading to abnormal proliferation of the vaginal microbiota and ascending infection, which ultimately results in PID manifestations including endometritis, pyometra, and tubo-ovarian abscesses.

## 5 Methods of physically inducing PID

### 5.1 Mechanical damage

The PID model is initiated through mechanical injury, primarily employed to facilitate the establishment of an infectious PID model alongside pathogens or chemical injuries (17, 63, 69–74, 77). Standard procedures involve the abrasion of the cervix or endometrium using sterile surgical instruments (17, 63, 69–74, 77), which compromises the mucosal barrier, elevates the risk of pathogen colonization, and increases the permeability to chemical damage agents, thereby promoting inflammatory responses. This approach is frequently combined with pathogen inoculation and chemical damage agents to enhance the model’s stability and reproducibility. For instance, a method combining mechanical injury with mixed bacterial exposure is utilized (17, 63, 69–74, 77). In the context of chemical induction, mechanical injury is less commonly employed, primarily due to the potential exacerbation of tissue damage resulting from chemical stimulation. Nonetheless, the potential for combined use to amplify the inflammatory response in specific experimental designs remains a consideration.

Mechanical injury is a critical step in establishing rat models of PID and Sequelae of Pelvic Inflammatory Disease (SPID). Its core mechanisms of action can be summarized in three points: First, it directly disrupts the physical barrier and intercellular junctions of the endometrial epithelium, creating pathological gaps that facilitate the adhesion, colonization, and deep invasion of pathogens such as *Escherichia coli* and *Staphylococcus aureus*, thereby significantly enhancing the stability and success rate of model establishment (69, 73); Second, by releasing DAMPs, it synergizes with pathogen-associated molecular patterns (PAMPs) from pathogens to activate core inflammatory pathways such as NF-κB, driving the cascading release of pro-inflammatory factors like IL-1*β* and TNF-*α* as well as the infiltration of inflammatory cells. This creates a vicious cycle of “barrier damage – infection exacerbation – inflammation amplification,” propelling acute PID into chronic inflammation (73, 74); Third, in conjunction with persistent chronic inflammation, it initiates abnormal tissue repair processes, inducing post-PID sequelae such as pelvic adhesions and fibrosis via the TGF-β/MMP-2 pathway. Simultaneously, it disrupts the balance of estrogen and progesterone receptor expression in the endometrium and inhibits the LIF/JAK2/STAT3 pathway—a key pathway for endometrial receptivity—thus fully replicating the pathological characteristics of the entire disease course of PID, from acute infection to reproductive dysfunction (69, 73).

### 5.2 Foreign body implantation

Finally, the introduction of foreign objects into the uterine cavity can elicit pelvic inflammatory responses. This method aims to provoke a localized chronic inflammatory reaction by implanting an inert plastic tube into the uterus (92). The procedure is detailed as follows (92): Female SD rats were selected and anesthetized via intraperitoneal injection of pentobarbital sodium. A midline abdominal incision, approximately 2 cm in length, was made to expose the left uterine horn. A transverse incision was then performed 1 cm from the anterior end of the uterine horn. An alcohol-sterilized plastic tube, measuring 2 mm in diameter, 0.5 cm in length, and weighing 5 mg, was inserted into the uterine cavity, secured with sutures, and the abdominal cavity was closed in layers. The entire procedure was conducted under aseptic conditions, and the animals were provided with normal feeding post-operation. The complete model construction process, from anesthesia induction to the conclusion of the operation, requires approximately 20–30 min. Following the procedure, the efficacy of the model was confirmed through the assessment of the uterine index and the quantification of inflammatory factor levels. It is evident that this approach is fundamentally distinct from the hormone intervention model and closely resembles the chronic inflammatory response induced by intrauterine devices, thereby offering a dependable method for investigating foreign body-mediated immune rejection and the local inflammatory microenvironment. Although the incidence of PID associated with intrauterine devices is relatively low (93–97), this model nonetheless serves as an effective platform for simulating chronic inflammation resulting from the persistent stimulation by foreign bodies.

The core mechanism by which the foreign body implantation method establishes a rat model of chronic PID involves precisely replicating clinical disease characteristics through a dual pathological cascade (94): mechanical damage to the pelvic reproductive organs caused by surgical procedures disrupts the mucosal barrier and triggers acute inflammation, recruiting innate immune cells and releasing pro-inflammatory mediators such as IL-1 and IL-6; The implanted non-degradable foreign body, acting as a persistent exogenous stimulus that cannot be cleared by the body, continuously activates pro-inflammatory signaling pathways, disrupting the pro-inflammatory–anti-inflammatory immune balance. This causes acute inflammation to progress into chronic granulomatous inflammation, while simultaneously inducing tissue fibrosis and pelvic adhesions. Furthermore, it can lead to the formation of bacterial biofilms, resulting in resistance to antibiotic treatment. This model fully aligns with the pathophysiology and therapeutic challenges of clinical chronic PID.

## 6 Integrated method-induced PID

Through the integration of diverse methodologies, including pathogen induction, chemical stimulation, and mechanical injury, our understanding of the current landscape of PID model construction, as well as the specific operations and applications of various induction techniques, has been enhanced. An examination of these methods reveals that the combined use of multiple induction techniques can substantially enhance the stability and pathological fidelity of the model. Consequently, the construction of PID models increasingly favors a multi-factor composite induction approach. For instance, the combination of pathogen infection with mechanical injury (17, 63, 69–74, 77), and the integration of pathogen induction with chemical stimulation (33), are noteworthy examples. In the approach involving pathogen infection coupled with mechanical injury, the majority of studies employ a mixed bacterial solution of *Escherichia coli* and *Staphylococcus aureus* in conjunction with mechanical injury. Additionally, some studies incorporate other pathogenic bacteria, such as *Ureaplasma urealyticum* and *Streptococcus pyogenes*, to further refine the model (17, 63, 69–74, 77). In a study utilizing the method of pathogen combined with chemical stimulation (33), researchers successfully developed a CPID model that closely resembles clinical manifestations. This was achieved through the use of a combination of *Escherichia coli*, *Staphylococcus aureus*, *Candida albicans*, and 7% phenol mucilage. The lower concentration of phenol mucilage employed in this method is attributed to the synergistic application of these pathogens. Furthermore, composite models incorporating bacteria with non-specific inducing factors, such as fatigue and hunger stress, have also been developed. By diminishing the body’s immunity to facilitate the persistent presence of infection, these models more accurately replicate the pathological characteristics of recurrent and CPID (29).

## 7 Discussion

Presently, PID continues to pose a significant threat to women’s reproductive health globally, necessitating further investigation into its pathogenesis and the refinement of prevention and treatment strategies. In PID model development, methodological innovations have evolved from single pathogen induction, chemical stimulation, and mechanical injury to the widespread adoption of multi-factor composite induction, reflecting a deeper understanding of PID’s pathological mechanisms. While the comparative performance of these methods across different experimental endpoints remains incompletely characterized, each offers distinct advantages for specific research scenarios and has substantial reference value for preclinical study design. Future research should prioritize establishing standardized composite induction protocols and elucidating dose–response relationships between pathogen combinations, stimulation intensities, and host responses.

However, this review has several limitations. First, the heterogeneity of animal models (e.g., species, strains, induction protocols) complicates cross-study comparisons and clinical translation. Second, most studies focus on acute inflammation, with limited data on chronic PID progression and long-term sequelae. Third, the lack of standardized evaluation metrics for model validity hinders consistent assessment of pathological fidelity. Fourth, the mechanisms underlying multi-pathogen synergism and host-pathogen interactions remain incompletely characterized, particularly regarding immune-metabolic crosstalk. Finally, the translational relevance of current models to human PID, especially regarding sexual transmission and hormonal influences, requires further validation.

## 8 Conclusion

In summary, appropriate animal models of PID are indispensable for exploring the pathogenesis of the disease and evaluating novel prevention and treatment strategies. Different modeling methods have their own characteristics and application scenarios: single-factor induction methods are suitable for studying the pathogenic role of a single specific factor, while multi-factor composite induction models can better simulate the complex multi-pathogenic environment of clinical PID, with higher stability and pathological similarity. At present, the research on PID modeling still has problems such as lack of standardized protocols, imperfect evaluation systems, and insufficient exploration of chronic lesions and pathogenic mechanisms. Future research needs to focus on addressing these limitations, constructing standardized, high-clinical-relevance PID animal models, so as to provide a more reliable experimental basis for in-depth research on PID and the development of new clinical intervention strategies.

## Glossary

PID: Pelvic inflammatory disease
Bax: Bcl-2-Associated X protein
Bcl-2: B-cell lymphoma-2
CD4^+^: Cluster of differentiation 4 positive
CD8^+^: Cluster of differentiation 8 positive
CHSP60: Chlamydial heat shock protein 60
COX-2: Cyclooxygenase-2
CXCL1/2: Chemokine (C-X-C motif) ligand 1/2
ECM: Extracellular matrix
ERK1/2: Extracellular signal-regulated kinase 1/2
Fas: Fas cell surface death receptor
FasL: Fas ligand
ICAM-1: Intercellular adhesion molecule-1
IFN-*γ*: Interferon-γ
IκB: Inhibitor of nuclear factor-kappa B
IκB-*α*: Inhibitor of nuclear factor-kappa B α
IKK: IκB kinase
IL-1β: Interleukin-1β
IL-4: Interleukin-4
IL-6: Interleukin-6
IL-8: Interleukin-8
IL-10: Interleukin-10
IL-17: Interleukin-17
JAK2: Janus kinase 2
JNK: c-Jun N-terminal kinase
LIF: Leukemia inhibitory factor
LPS: Lipopolysaccharide
LXA4: Lipoxin A4
MAPK: Mitogen-activated protein kinase
MMP-2: Matrix metalloproteinase-2
MMP-9: Matrix metalloproteinase-9
MMPs: Matrix metalloproteinases
MyD88: Myeloid differentiation primary response 88
NF-κB: Nuclear factor-kappa B
NF-κB p65: Nuclear factor-kappa B p65 subunit
NO: Nitric Oxide
p53: Tumor protein p53
PGE2: Prostaglandin E2
SD rats: Sprague–Dawley rats
Smad2/3: Mothers against decapentaplegic homolog 2/3
STAT3: Signal transducer and activator of transcription 3
TGF-*β*: Transforming growth factor-β
TGF-β1: Transforming growth factor-β1
Th1: T Helper 1 cell
Th2: T Helper 2 cell
TIMP-1: Tissue inhibitor of metalloproteinase-1
TLR: Toll-like receptor
TLR2: Toll-like receptor 2
TLR4: Toll-like receptor 4
TNF-*α*: Tumor necrosis factor-α
TRAF: TNF receptor-associated factor
VEGF: Vascular endothelial growth factor
VEGF-β: Vascular endothelial growth factor-β
